# Evolutionary History of the Live-Bearing Endemic *Allotoca diazi* Species Complex (Actinopterygii, Goodeinae): Evidence of Founder Effect Events in the Mexican Pre-Hispanic Period

**DOI:** 10.1371/journal.pone.0124138

**Published:** 2015-05-06

**Authors:** Diushi Keri Corona-Santiago, Ignacio Doadrio, Omar Domínguez-Domínguez

**Affiliations:** 1 Programa Institucional de Maestría en Ciencias Biológicas, Facultad de Biología, Universidad Michoacana de San Nicolás de Hidalgo, Morelia, Michoacán, México; 2 Laboratorio de Biología Acuática, Facultad de Biología, Universidad Michoacana de San Nicolás de Hidalgo, Morelia, Michoacán, México; 3 Departamento de Biodiversidad y Biología Evolutiva, Museo Nacional de Ciencias Naturales, CSIC, Madrid, España; University of Calgary, CANADA

## Abstract

The evolutionary history of Mexican ichthyofauna has been strongly linked to natural events, and the impact of pre-Hispanic cultures is little known. The live-bearing fish species *Allotoca diazi*, *Allotoca meeki* and *Allotoca catarinae* occur in areas of biological, cultural and economic importance in central Mexico: Pátzcuaro basin, Zirahuén basin, and the Cupatitzio River, respectively. The species are closely related genetically and morphologically, and hypotheses have attempted to explain their systematics and biogeography. Mitochondrial DNA and microsatellite markers were used to investigate the evolutionary history of the complex. The species complex shows minimal genetic differentiation. The separation of *A*. *diazi* and *A*. *meeki* was dated to 400–7000 years ago, explained by geological and climate events. A bottleneck and reduction of genetic diversity in *Allotoca diazi* was detected, attributed to recent climate fluctuations and anthropogenic activity. The isolation of *A*. *catarinae* occurred ~1900 years ago. No geological events are documented in the area during this period, but the date is contemporary with P’urhépecha culture settlements. This founder effect represents the first evidence of fish species translocation by a pre-Hispanic culture of Mexico. The response of the complex to climate fluctuation, geological changes and human activity in the past and the future according to the ecological niches predictions indicates areas of vulnerability and important information for conservation. The new genetic information showed that the *Allotoca diazi* complex consist of two genetic groups with an incomplete lineage sorting pattern: Pátzcuaro and Zirahuén lakes, and an introduced population in the Cupatitzio River.

## Introduction

The diversity and distribution of freshwater ichthyofauna has been strongly linked to natural events, mainly geological process and historical climatic fluctuations. It is therefore that, taking into account the biogeographic history of each region in the world; the freshwater fish have been used as a model for the study of biogeography, speciation, paleohydrology and evolution. However, translocation and introduction of fish species is well documented in many ancient cultures having an important role in their current distribution, misunderstanding the evolutionary history of the species, In America, particularly in Mexico, the impact of pre-Hispanic cultures in the current distribution of the biota, is little known and mostly documented for birds [1–3]. In fact, the scientific community has long assumed that translocations and introduction of species in America occurred only after European colonization [4].

The region in West-central Mexico, particularly between the Lerma-Santiago and Balsas rivers, is characterized by varied and rugged physiography, a product of intense tectonic and volcanic activity in the area since the Miocene [5]. Also, this region is an area with a long cultural history, with the P’urhépecha settlement developing one of the most stable empires at least 3000 years ago [6], particularly due to the richness and abundance of lacustrine resources. The main resources currently exploited by settlements around the lakes are fish, as well as turtles, salamanders, clams, and wetland plants [7,8]. Even the area was formerly known by the Náhuatl culture as Mechuacan, “a place of fishermen,” and is currently called Michoacán.

Approximately 100 fish species are in the West-central Mexico, 70% of which are endemic and one of the most representative endemic fish groups of this region is the subfamily Goodeinae [5]. The group presents high species richness in a small area (c. 41 species) and unique characteristics associated with breeding strategies and embryo development, such as internal fertilization, matrotrophy, and sexual selection [9–13]. The family presents a high diversification apparently influenced by viviparity, with vicariance and adaptive radiation being the most important factors. The speciation rate of goodeids has not been constant and has been impacted by multiple extinctions, estimating that at least 25 000 years is required to establish an evolutionary lineage [14].

**Fig 1:**
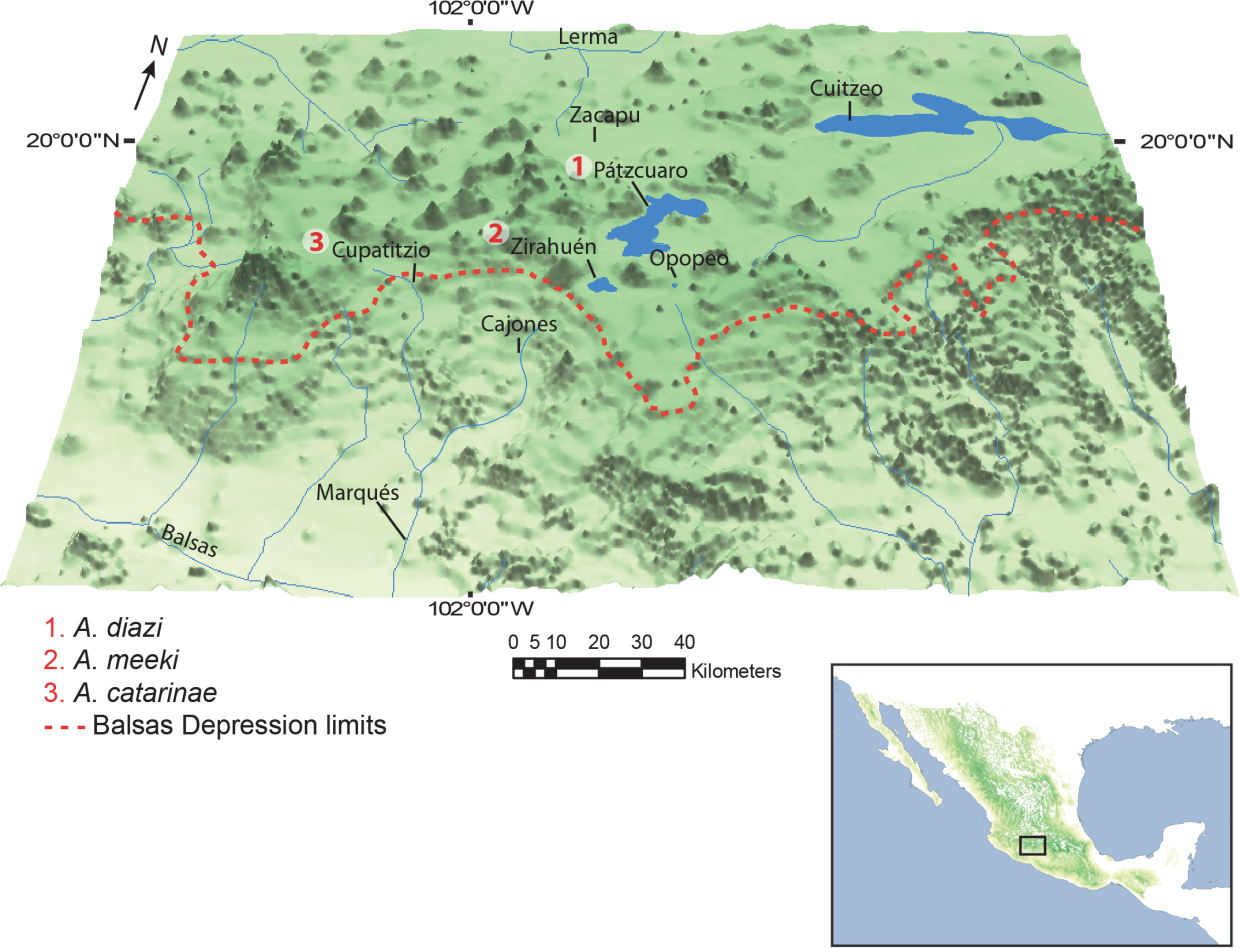
Geographical distribution of *Allotoca diazi* complex.

The genus *Allotoca* is the most diverse within the Goodeinae, and is the subject of taxonomic controversy, with the need for taxonomic revision to validate species [9,10,15–17]. The *A*. *diazi* complex comprises three recognized species [5]: *Allotoca diazi* [18], endemic to Pátzcuaro basin, *A*. *meeki* [16], endemic in the Zirahuén basin, and *Allotoca catarinae* [15], restricted to the Cupatitzio River, an upper tributary of the Balsas River basin (Fig 1). *Allotoca diazi* was extirpated from Pátzcuaro Lake and is currently restricted to the Chapultepec Spring, a tributary of Pátzcuaro Lake, while *A*. *meeki* was extirpated from Zirahuén Lake and currently restricted to Opopeo Spring, a tributary of the Zirahuén Lake [5]. The lakes are located in the Michoacán-Guanajuato Corridor, an area of over 1000 volcanic cones active from the Pliocene to the present. To explain the evolution of these three sister species and other co-distributed taxa, two biogeographic hypotheses have been proposed. The first argues the existence of a tributary connecting the Lerma River with Cuitzeo, Pátzcuaro, and Zirahuén lakes [19] around 700 000 years ago [20]. The second hypothesis indicates the existence of a tributary connecting the Cupatitzio River with Zirahuén and Pátzcuaro lakes, reaching Zacapu [21].

Additionally, the application of modern biogeographic, genetic, systematic, and bioinformatics methodologies in the study of evolutionary processes within the Goodeinae has questioned recent connections of the Lerma and Cupatitzio Rivers with the Zirahuén, Pátzcuaro, Cuitzeo, and Zacapu basins [9,12,22,23]. The taxonomic status of the three species has also been questioned [13,24], and the possibility of pre-Hispanic translocation of exploited species has been proposed [12].

In order to elucidate and analyze the two biogeographic hypotheses about the connections or disconnections between the Zirahuén, Pátzcuaro and Cupatitzio basins, and the possibility of a species translocation, we examined the evolutionary history of *Allotoca diazi* complex using two different molecular markers described for the Goodeinae Subfamily [9,25,26]: a conserved molecular marker of mitochondrial DNA (Cytochrome *b*, Cyt*b* gene) and a less conserved microsatellite nuclear markers. We obtained these molecular markers to explore the genetic differentiation at intraspecific and interspecific level applying phylogeographic and phylogenetic approaches. The results of the genetic study were analyzed based on geological, paleoclimatic and anthropogenic records from central Mexico to access how natural and artificial historical processes are involved in the evolutionary history of *A*. *diazi* complex. In addition, we used all the genetic information and ecological niche predictions for identify operational conservation units.

## Materials and Methods

### Ethics statements

This research was a part of the multidisciplinary work for restoration and conservation of the Cupatitzio River, and the complete work, field and laboratory protocols were approved by the Mexican Government, SEMARNAT (The Ministry of Environment and Natural Resources for Mexico) and CONACyT (National Council of Sciences And Technology) (Permit No. FOMIX-MICH-2009-C02-115897). All individuals were anesthetized using Tricaine mesylate (MS-222) to minimize suffering.

### Fish sampling and DNA isolation

One hundred and twenty specimens were collected from six populations throughout the distribution range of the species complex (S1 Table). Our sampling did not recover any *A*. *diazi* or *A*. *meeki* from Pátzcuaro Lake and Zirahuén lacustrine basin, respectively. This is consistent with reports that these species were extirpated from these basins as a result of the introduction of exotic species [5]. Capture was by electrofishing between March and August 2011. Pectoral or caudal fin clips, were obtained and preserved in absolute ethanol and frozen at -20°C. Some specimens of the species complex were identified and deposited in the fish collection of the Universidad Michoacana de San Nicolás de Hidalgo CPUM in México; the remaining individuals were returned to the water. Approximately 2 mm^2^ of tissue was used for DNA extraction. Digestion was performed with ATL QIAGEN Buffer and Proteinase K and purifying by BioSprint DNA Blood Kit QIAGEN according to the manufacturer’s instructions.

### Locus amplification, sequencing, and genotyping

Polymerase Chain Reaction (PCR) was performed to amplify the molecular markers. To amplify the Cyt*b* gene, we used the primers GLuDG [27] and H16460 [28]. The PCR consisted of a 12 μl volume reaction with a final concentration of 0.2 μM of each primer, 0.25 mM of each dNTP, 1.5 M of MgCl_2_, and 1 unit of *Taq* DNA Polymerase. The PCR procedure consisted of 2 min at 94°C followed by 35 cycles of 45 s at the 94°C for DNA denaturation, 1 min at 46°C for primer alignment, 1.5 min at 72°C for synthesis, and a final extension of 5 min at 72°C. The PCR products were checked by electrophoresis in agarose gel 1.5% and submitted to Macrogen Inc. (Korea) for sequencing.

For microsatellites, we searched polymorphic loci of the *A*. *diazi* complex using 20 primers designed for Goodeinae [25,26]. Seven polymorphic microsatellite loci were selected (S2 Table). The 7 loci were amplified with QIAGEN Multiplex PCR Kit according with the manufacturer specifications, and the products were submitted for genotyping (Secugen S. L. Corp, Spain).

### mtDNA analysis

Manual alignment was implemented in Mega v5.2 [29] and examined chromatographically. The genetic diversity was estimated by calculating nucleotide diversity (π), haplotype diversity (h) and proportion of segregating sites (Θ) using the software DNAsp v5.0 [30]. Neutrality tests (Tajima’s *D* and Fu’s FS) to Cyt*b* were implemented using Arlequin v3.5.1.3 [31]. The genetic *P*-distances separating the three species were obtained (D_*p*_) with Mega v5.2, and a bootstrapping process was implemented with 1000 repetitions. We conducted an analysis of geographic correspondence by network haplotype reconstruction using Network v4.6.1.0 [32], applying the median-joining method.

Tree-building algorithms were used for phylogenetic reconstruction using *Allotoca zacapuesis*, a sister group of the species complex [9], as outgroup. Neighbor-joining clustering was implemented in Mega v5.2 based on D_*p*_ with bootstrapping with 1000 replicates. Maximum likelihood reconstruction was conducted with RAxMLGUI v1.3 [33,34], using the generalized time reversible (GTR) + gamma + proportion of invariable sites model [33] and 1000 bootstrap repetitions. The evolutionary substitution model selected was the transitional model [35] + invariable site proportion + gamma (TMI+I+G) using jModeltest v1.7 [36] and selected by the Akaike information criterion (AIC) (S3 Table). Phylogenetic reconstruction was by Bayesian inference (BI) with the software MrBayes v3.1.2 [37], using the above selected evolutionary substitution model and implementing 2 reactions for 4 Markov Chains Monte Carlo (MCMC) processes with 3 million generations, sampling every 100 generations. We evaluated the convergence of the log-likelihood (-InL) value of the 2 reactions, with 10% of reconstructions discarded as burn-in to construct the consensus tree (σ = 0.0002). The posterior probabilities obtained based on a confidence limit of 95% (highest posterior density-HPD) were used to evaluate the support values of nodes.

### Microsatellite analysis

Quality of the samples genotyped and polymorphism in microsatellite loci was analyzed using GeneMapper v 4. Allele number by loci and species (N_a_), effective number of alleles (N_ae_), null allele proportion, and linkage disequilibrium were obtained using Arlequin v3.5.1.3 and Genepop v4.1.2 [38]. Observed (H_o_) and expected (H_e_) heterozygosity was determined, and the endogamy coefficient was obtained based on Wright’s fixation index (F_IS_) [39] to calculate the Hardy-Weinberg (HW) equilibrium across each locus and species using the software Arlequin v3.5.1.3, implementing 100 000 permutations.

The genetic structure level among the three species of the *A*. *diazi* complex was estimated with paired fixation indices (F_ST_). Significance was assessed by AMOVA with 20 000 permutations implemented in the software Arlequin v 3.5.1.3 using multiple hypothetical arrangements based on phylogenetic and biogeographic information. A Bonferroni correction [40] was applied to each *P*-value obtained in the paired test, HW analysis and linkage disequilibrium estimation. Bayesian clustering was conducted with the software Structure v2.3.3 [41]. We performed a series of independent runs from values of K = 1–10 populations assuming correlated allele frequencies and an admixture model [42] with 10 runs for each K value to assess convergence of results without geographic information. For each K value, the MCMC was run with a burn-in of 100 000 steps and chain length of 500 000. Following the method described by Evanno *et al*. 2005 [43], ΔK was calculated to find the K value that best explained the genetic differentiation level.

To determine whether the difference in allele size contributed to genetic structure and if each locus is consistent with a strict Stepwise Mutation model, we estimated the R_ST_ values to assess deviation of equilibrium between R_ST_ and F_ST_ values. R_ST_ values greater than F_ST_ imply influence of the size differences of alleles on interpopulation genetic differentiation; thus we can assume that loci mutate faster by multi-step changes [44–47]. The R_ST_ estimation was performed with SPAGeDi v1.3a [48] without geographic information, applying 20 000 permutations to obtain a test of differences among observed and expected R_ST_ and F_ST_ values.

### Isolation-with-migration and demographic history analyses

This approach allows determination of gene flow before and after isolation within a population and assesses phylogenetic relationship scenarios in recent isolation events within closely related populations or species. Divergence times of populations of members of the *A*. *diazi* complex were calculated using three analyses in IMa2 software [49], which has the advantage of implementing models of gene flow.

Pairwise analysis was conducted to find the confidence limits of the time to divergence among the three species, combining the molecular markers to substantiate and support the exchange rates within Markov chains. A molecular clock was calibrated using the mutation rate for Cyt*b* estimated for teleosts of 0.76–2.2%/million years (4.33*10^-6^-1.25*10^-5^subst/locus/generation) [50–52] and the Hasegawa-Kishino-Yano (HKY) evolutionary substitution model [53]. The mutation rate used for calibration with microsatellites was 0.9*10^-4^-1.5*10^–4^ locus/gamete/generation, estimation obtained for the Zebra fish *Danio rerio* [54] and congruent with the mutation rate for vertebrates [55,56]. The SMM evolutionary model was implemented for microsatellites. Ten independent runs were conducted for each scenario using 150 Markov chains with a burn-in of 500 000 for a total of 5 000 000, sampling topologies every 10 000 steps to recover 100 000 topologies from all runs. It was assumed that effective population size and the migration rate was different in each period.

The demographic history of the members of the species complex was obtained constructing Bayesian skyline plots (BSP) using Beast 1.8.0 [57] for Cyt*b* marker. Chains were run for 70 000 000 steps sampling each 1000 and 10% was discarded as burn-in under HKY substitution model, a relaxed molecular clock calibrated with the information of Cyt*b* mentioned above. All operators were optimized automatically and the results were analyses and visualized using Tracer 1.5 [58]. Effective sample size (ESS) was used to evaluate the strength of the analyses, and we determined the marginal distributions and HPD values in both analyses.

A test to identify recent bottlenecks was conducted using Bottleneck v1.2.0.2 [59] with three evolutionary models for microsatellites: Infinite Allele Model (IAM) [60], Two-Phase Model [61], and Stepwise Mutation Model (SMM) [62]. The test was implemented with 10 000 iterations based on a confidence limit of 95%. A mode-shift test was conducted to detect significant deviations in the allelic frequencies in populations that have experienced a recent bottleneck [63].

### Identification of operational conservation units and predictions of future distribution

The genetic, biological, and ecological information obtained in this investigation, along with relevant published information [64,65], was used for identification of conservation units. In order to predict a future scenario for range of distribution of the natural members of the species complex, a discriminant analysis of presence-only data through maximum entropy using the program Maxent v3.3.3 [66–67] was conducted for the present and for two periods in the future, years 2041 to 2060 and 2061 to 2080. The 6.0 Representative Concentration Pathways (RCP6.0) [68] was selected from downscaling Intergovernmental Panel on Climate Change 5 scenario (IPPC5) (Coupled Model Intercomparison Project 5) [69] implementing the Community Climate System Model 4 (CCSM4) [70]. We implemented independent runs of 5000 iterations with 19 climatic variables (http://www.worldclim.org/bioclim), bootstrapping of 100 replicates to obtain 95% confidence intervals, regularization multiplier (RM) values from 0.01 to 10, evaluating area under the ROC curve (AUC) values and using the omission rates to discard identical AUC of different RM values. Collinear climatic variables were discarded (S4 Table).

## Results

### mtDNA analysis

**Fig 2:**
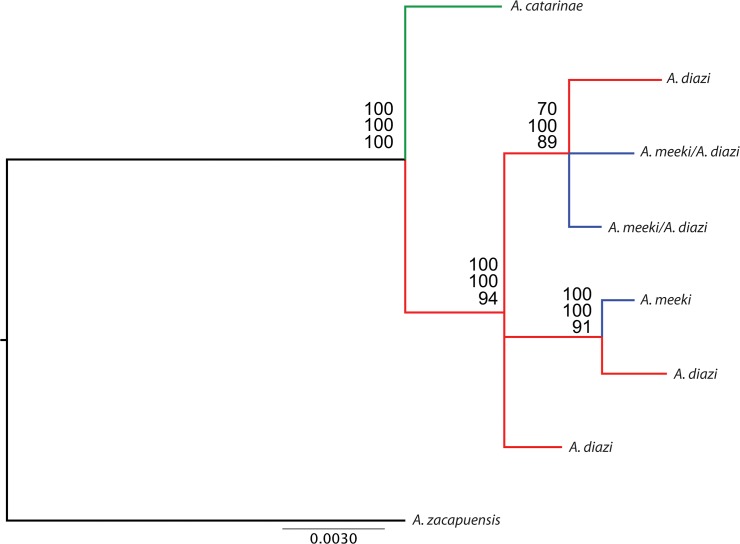
Phylogenetic inference based on haplotypes using Neighbor-joining, **Maximum likelihood and Bayesian inference of Cyt*b* gene.** Red asterisk: *Allotoca diazi* (n = 35 Hn = 5), Blue asterisk: *A*. *meeki* (n = 22 Hn = 3) and Green asterisk: *A*. *catarinae* (n = 52). Support values are represented by numbers above the nodes for analyses in the order mentioned above.

**Fig 3:**
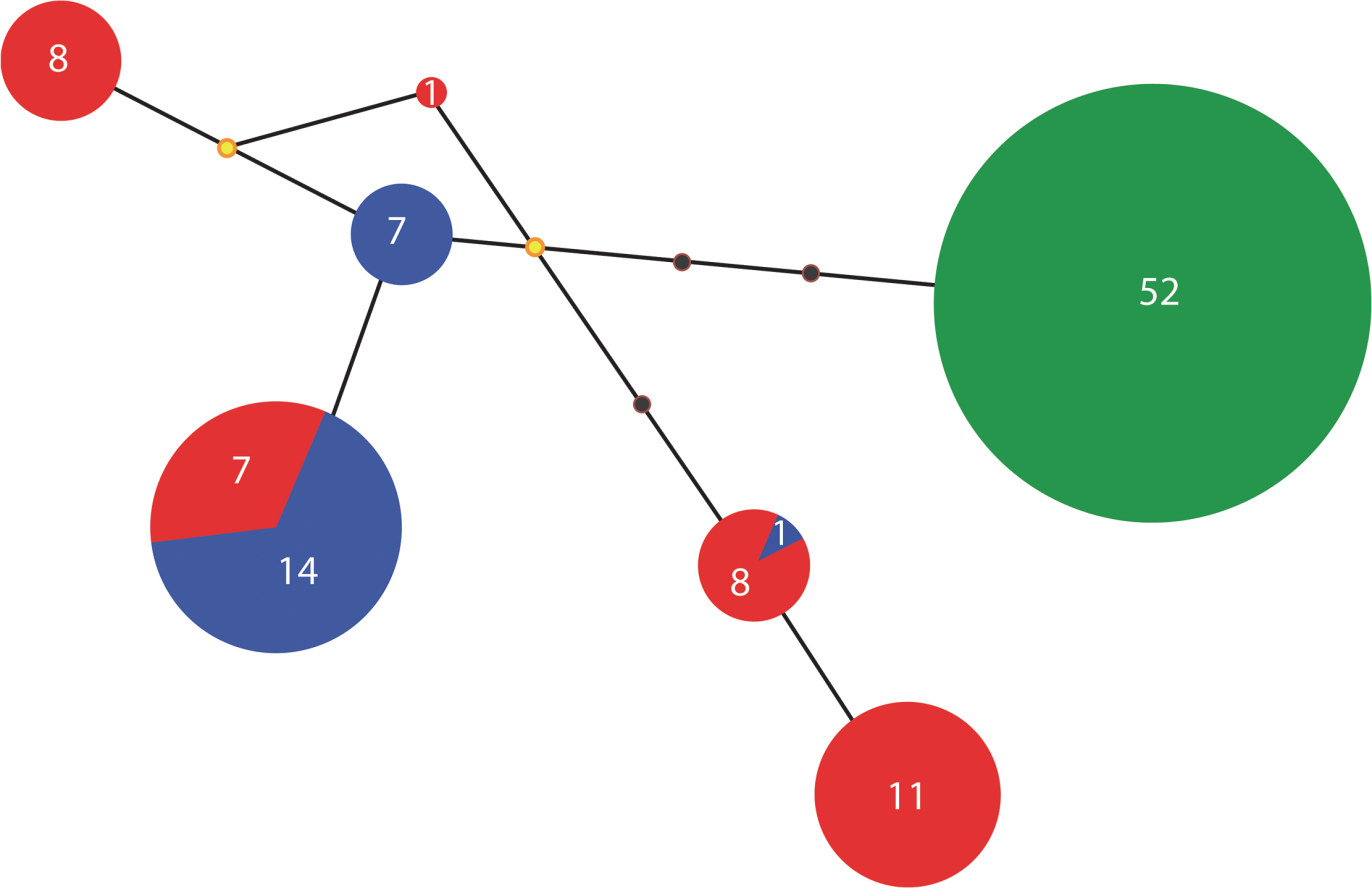
Haplotype network based on Cyt*b* gene: Red asterisk: *A*. *diazi* (n = 35 Hn = 5), Blue asterisk: *A*. *meeki* (n = 22 Hn = 3) and Green asterisk: *A*. *catarinae* (n = 52). Values represent the number of individuals per haplotype. The yellow circles represent the median-vectors.

One hundred nine sequences of the mitochondrial gene Cyt*b* (1037pb) were obtained for the sampled populations of the three species (GenBank accession numbers: KJ776467-KJ776575) (S1 Table). Overall, *A*. *diazi* and *A*. *meeki* nucleotide diversity was 0.0005±0.0002, haplotype diversity was 0.65±0.13, and mean proportion of segregating sites per locus was 1.4±0.3. *Allotoca catarinae* showed null genetic diversity in four sampled populations with a unique haplotype. *Allotoca diazi* was the most genetically diverse with five haplotypes (n = 35), followed by *A*. *meeki* with three haplotypes (n = 22), of their respective and unique populations (Table 1). No significant deviation from neutrality was found for the *A*. *diazi* complex. The mean of the genetic distances of *A*. *catarinae* with respect to *A*. *diazi* and *A*. *meeki* was 0.6% and between *A*. *diazi* and *A*. *meeki* was 0.3%. The three phylogenetic approximations were congruent. The topologies were similar with all DNA sequences and haplotypes. The tree based on haplotypes is presented in Fig 2. The consensus tree was formed by two haplogroups: a non-monophyletic group of *A*. *diazi*/*A*. *meeki* and a group with the complete of *A*. *catarinae*. The haplotype network recovered the same two genetic groups, with four mutational steps separating them (Fig 3). Spatial congruence was not found in haplogroup *A*. *diazi*/*A*. *meeki*. Two haplotypes of *A*. *diazi* were shared with *A*. *meeki*, and the unique haplotype of *A*. *catarinae* is not shared with the other members of the species complex.

**Table 1:** Genetic diversity parameters and neutrality test based on Cyt*b* gene sequences.

Species	n	Hn	Populations	π	h	Θ_S_	Θ_L_	FS	D_T_
*A*. *diazi*	35	5	1	0.0030	0.78	0.0016	1.70	2.07ns	1.61ns
*A*. *meeki*	22	3	1	0.0007	0.52	0.0010	1.10	0.69ns	-0.87ns
*A*. *catarinae*	52	1	4	0	0	0	0	-	-

n = sample size, Hn = haplotype number, π = nucleotide diversity, h = haplotype diversity, Θ_S_ = proportion of segregating sites per site, Θ_L_ = segregate sites per locus, FS = Fu’s FS test, D_T_ = Tajima’s *D* test, ns = no significant deviation of neutrality test (*P*>0.05).

### Microsatellite analysis

For the 7 microsatellites loci amplified in populations of the *A*. *diazi* complex, we obtained a total of 96 genotypes. The average number of alleles per locus was 10.4±3.5; the number of genetic variants contributing to heterozygosity in each locus of the species complex was 5.8±2.2; and the proportion of null alleles was 0.06±0.03 (*P* > 0.05). Significant deviation from linkage equilibrium was not found at most loci, LD = 0.06±0.03 (*P* > 0.05). Significant linkage disequilibrium was observed in two loci of *A*. *diazi* (S5 Table). Significant deviation from HW equilibrium associated with lower than expected heterozygosity was found in *A*. *diazi* and *A*. *catarinae* (*P* < 0.05) (Table 2).

**Table 2:** Genetic diversity, HW equilibrium, and bottleneck test for microsatellite loci.

Species	Locus	H_o_	H_e_	HW	F_IS_	BNK IAM/TPM/SMM
*A*. *diazi*	XC18	0.84	0.86	—	0.141	-/-/*
	ZT1.6	0.84	0.78	—		
	ZT1.7	0.73	0.88	<0.001		
	IW196	0.62	0.76	<0.05		
	XC25	0.30	0.46	<0.001		
	AS2	0.60	0.81	<0.001		
	ZT1.9	0.70	0.83	<0.05		
*A*. *meeki*	XC18	0.83	0.90	—	0.088	*/-/-
	ZT1.6	0.78	0.92	—		
	ZT1.7	0.89	0.88	—		
	IW196	0.72	0.88	—		
	XC25	0.67	0.67	—		
	AS2	0.78	0.88	—		
	ZT1.9	0.83	0.90	—		
*A*. *catarinae*	XC18	0.71	0.89	<0.001	0.151	*/-/*
	ZT1.6	0.66	0.74	—		
	ZT1.7	0.81	0.87	<0.05		
	IW196	0.59	0.61	—		
	XC25	0.22	0.20	—		
	AS2	0.59	0.79	<0.001		
	ZT1.9	0.66	0.93	<0.001		

H_o_ = observed heterozygosity; H_e_ = expected heterozygosity; HW = Hardy Weinberg deviation test; FIS = endogamy coefficient (Bold letters-*P*<0.05). Identifying bottleneck (BNK) with Wilcoxon test (*P*<0.05) based on infinite allele model (IAM), two phases model (TPM), and stepwise model (SMM) (* significant;—non-significant). The *P*-values were corrected with Bonferroni method.

**Fig 4:**
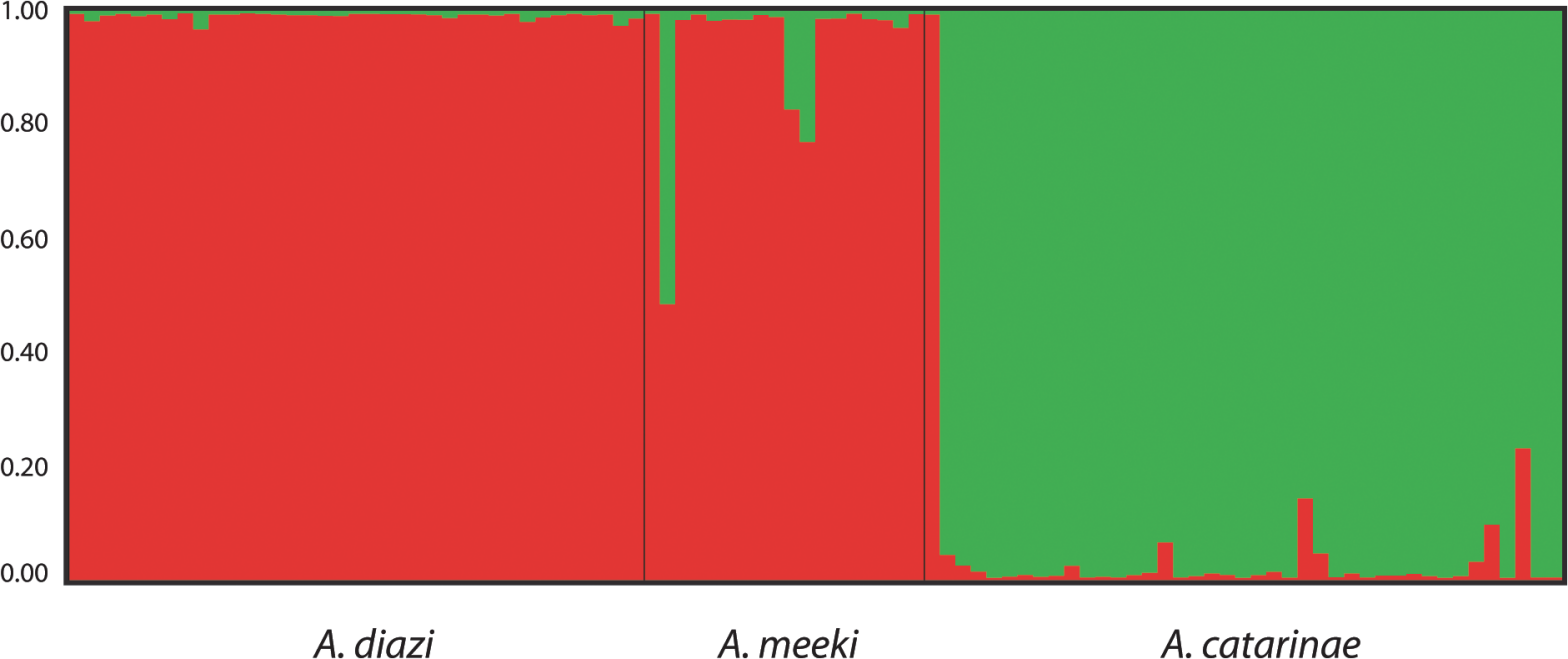
Genetic structure by Bayesian inference based on microsatellite loci.

The observed R_ST_ values for all loci were not significantly different from expected (Ho:R_ST_ = *p*R_ST_) or from the F_ST_ values (S6 Table), suggesting that allele size difference did not contribute to the genetic structure of the *A*. *diazi* complex. Low but significant genetic differentiation was detected (F_ST_ = 0.113 *P* < 0.05) via AMOVA analysis (S7 Table). Genetic structure by BI recovered two genetic groups (*A*. *diazi*+*A*.*meeki—A*. *meeki*) with a high proportion of individuals assigned to other groups (0.3–0.7) (Fig 4). However, significant genetic differentiation was detected between the three members of the species complex. The lowest F_ST_ value of genetic differentiation was found between *Allotoca diazi* and *A*. *meeki* (0.084), while genetic differentiation of *A*. *catarinae* from *A*. *diazi* and *A*. *meeki* was 0.133 and 0.109 (*P*<0.05), respectively (Table 3). The number of migrants value between members of the species complex were not significant.

**Table 3:** Estimated genetic differentiation based on microsatellites.

	*A*. *diazi*	*A*. *meeki*	*A*. *catarinae*
*A*. *diazi*	—	5.5	3.3
*A*. *meeki*	0.084*	—	4.1
*A*. *catarinae*	0.133*	0.109*	—

Pairwise F_ST_ values (under the diagonal) and number of migrants (Nm) (above the diagonal).

* Significance value *P*<0.05 after Bonferroni correction.

Genetic structure by BI recovered two genetic groups (K = 2 *A*. *catarinae* + *A*. *diazi/A*.*meeki*) with high proportion of individuals assigned to another group (0.3±0.7) (Figs 3 and S1).

### Estimate of divergence times and demographic history

The *A*. *diazi* complex divergence from its common ancestor *A*. *zacapuensis* was estimated to have occurred in the past million years. The isolation event of *A*. *diazi* from *A*. *meeki* occurred in the past 7000–400 years (HPD≥95%) with the peak of probability estimated as 1700 years ago (Table 4 and S2 Fig). Divergence time and isolation of *A*. *catarinae* from *A*. *diazi* and *A*. *meeki* was calculated at 8000–700 years ago, with the highest probability being 1900 years ago for *A*. *diazi* and 2500 years ago for *A*. *meeki*.

**Table 4:** Divergence times based on mitochondrial Cyt*b* gene and microsatellite loci in an Isolation-with-Migration model.

	HiPk years	Divergence times years (HPD)	2Nm
*A*. *diazi*			
*A*. *diazi/A*. *meeki*	1702	414–6760	0.0–1.8ns
*A*. *meeki*			
*A*. *diazi/A*. *catarinae*	1886	690–8048	0.3–1.4ns
*A*. *catarinae*			
*A*. *meeki/A*. *catarinae*	2529	830–8030	0.4–1.4ns

HiPk = highest peak ns = no significance (*P* > 0.05); number of migrants (Nm); Highest Posterior Density (HPD) interval.

The migration rate estimated among the members of the *A*. *diazi* complex was fewer than 5.5 individuals/generation and was not significant (Table 3). BSP analysis using Cyt*b* revealed demographic decline in the last 10 000 years for *A*. *catarinae* (HPD≥95%) (S4 Fig), and recent bottlenecks for the three members of the species complex were detected out of the HPD confidence range. However, recent bottlenecks were identified in the three species based on different evolutionary models using microsatellites. The bottlenecks detected in *A*. *diazi* and *A*. *catarinae* via SMM were supported by the F_IS_ coefficients: 0.141 and 0.151, respectively. The bottleneck detected in *A*. *meeki* was associated with higher than expected heterozygosity and allele size differences in the IAM analysis.

### Operational conservation units and predictions of future distribution

We included in the analysis the species considered as natural members of the species complex: *Allotoca diazi* and *A*. *meeki*. The species *Allotoca catarinae* was excluded of the analysis because could be result of translocation. The predictions were performed taking into account that the gene flow between species will be null.

**Fig 5:**
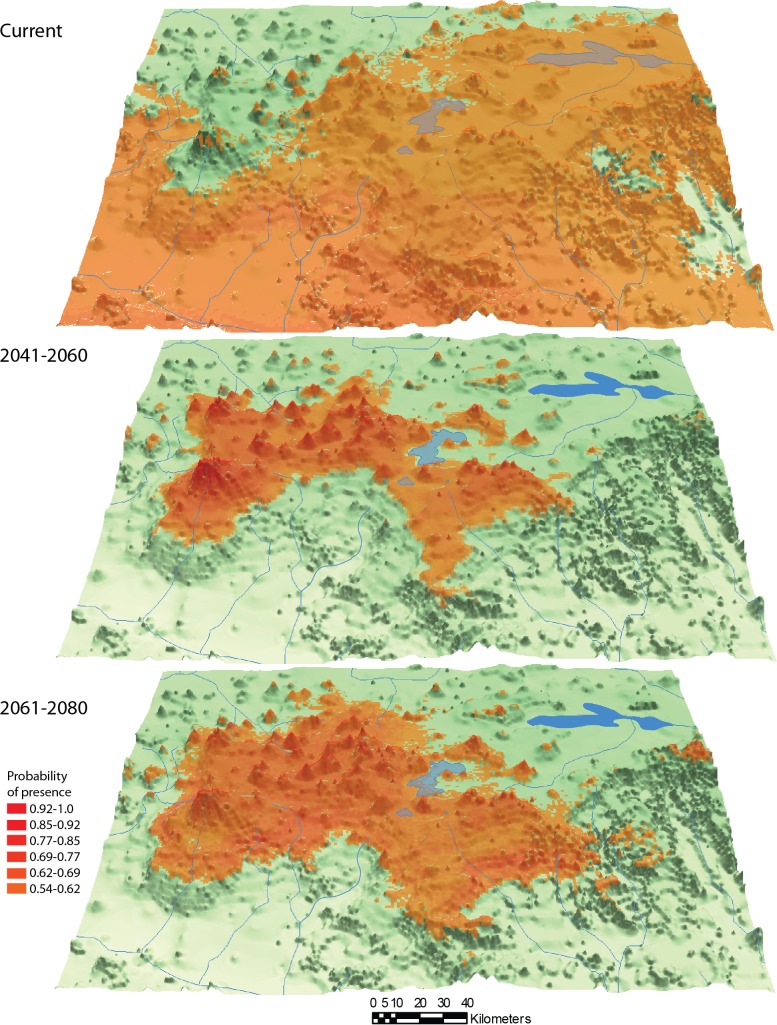
Ecological niche projections for *Allotoca diazi* in present and two future periods.

**Fig 6:**
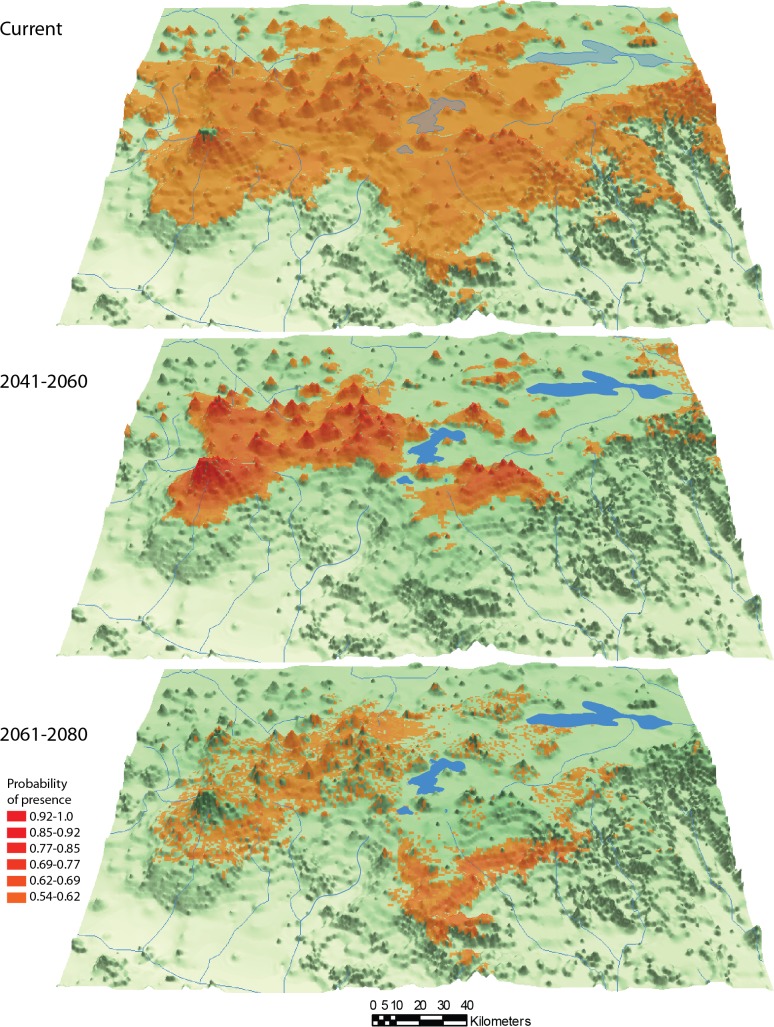
Ecological niche projections for *Allotoca meeki* in present and two future periods.

The ecological niches area predicted with present environment conditions for *Allotoca diazi* and *A*. *meeki* is bigger than their real current distribution. In the case of the ecological niche models for two periods in the future, high AUC values for were obtained for both species (AUC = 0.940–0.995 RM = 0.01–0.1, see S4 Table). In the first period between the years 2041 to 2060, the model suggest that the ecological niches available for the two species will be located at higher altitudes than they are currently distributed (Figs 5 and 6). In the second period of the future (2061–2080), the area of ecological niches predicted will increase for *A*. *diazi* but the probability of occurrence in the Pátzcuaro Lake is low. In the same period, the ecological niches predicted to *A*. *meeki* could decrease drastically. According with the genetic and future predictions, we propose the existence of two OCUs corresponding to natural populations in Pátzcuaro and Zirahuén basins.

## Discussion

### *Allotoca diazi* complex

The isolation of the *Allotoca diazi* complex from its common ancestor *A*. *zacapuensis*, does not contradict the hypothesis of Álvarez (1972) [21] with respect to the connection between the Zacapu and Pátzcuaro basins during the Pleistocene, resulted from the formation of the El Zirate mountain and the northern Pátzcuaro Lake shoreline during the Late Pleistocene [71].

The *Allotoca diazi* complex consists of a genetic group with low genetic structure and its isolation within the members was more recent, during the Holocene. The isolation of *A*. *diazi*, *A*. *meeki*, and *A*. *catarinae* is reflected in the low genetic differences, non-monophyletic patterns, shared haplotypes, and genetic groups assignment, and we considered that the species complex consist in an incomplete lineage sorting pattern [72–75]. The genetic distances among the three species in Cyt*b* are smaller than reported to sister Goodeinae species (1.7–11%) [9] and sister species, congeneric species, and cofamilial genera within and across the major vertebrate taxonomic classes (~2%) [76–79]. The genetic variation estimated in the species complex based on the Cyt*b* gene is attributed to genetic drift and mutation (D_T_ and FS values did not show significant deviation from neutrality). Low genetic variation was detected as a genetic pool arrangement through AMOVA analysis. However, BI detected this minimum mark of genetic structure as two genetic groups. The results obtained are supported by the minimal morphological differences previously reported [15,17,80–84].

### *Allotoca diazi* and *A*. *meeki* isolation

The hypothesis of connection and disconnection of Pátzcuaro and Zirahuén lakes during the last 700 000 years [19] is supported by stratigraphic [85]; limnologic data [86,87]; shared native icthyofauna such as *Chirostoma estor*, *Chirostoma pátzcuaro* (Atherinopsidae), *Algansea lacustris* (Cypronidae), *Alloophorus robustus*, *Skiffia lermae*, *Allotoca dugesii*, and *Goodea atripinnis* (Goodeidae) [88]; and genetic data [12,89].

Shared haplotypes of *A*. *diazi* and *A*. *meeki* and the high proportion of individuals assigned to other species (0.5 to 0.7) by microsatellite data, suggest recent gene flow. Microsatellite information showed low level of genetic differentiation (F_ST_ = 0.084). Low genetic diversity was estimated for *A*. *diazi* in the Cyt*b* gene (h = 0.78, π = 0.0030) and even lower for *A*. *meeki* (h = 0.52, π = 0.0007). The migration rate between the two species is not significant any time.

We estimated that the isolation of *A*. *diazi* from *A*. *meeki* occurred in the past 400–7000 years, which is consistent with geological, climatic, and anthropogenic events involving Pátzcuaro and Zirahuén lakes. Tecto-volcanic events giving rise to the separation of Pátzcuaro and Zirahuén lakes began 8000 years ago with the formation and activity of the La Tasa volcano southwest of Lake Pátzcuaro and climate fluctuations causing decline in water level and drying of streams [21,90–93]. Fluctuations in the level of Lake Pátzcuaro during the Holocene associated with climate change, human activity [90,91,94–99], and occurrence of tsunamis [100] could be involved to the isolation and demographics changes of *A*. *diazi* and *A*. *meeki*. These demographic changes were reflected in genetic diversity including the positive D_T_ value, which was found to be low in *A*. *diazi*, and associated with recent bottlenecks that promoted inbreeding (F_IS_ = 0.141), reduced heterozygosity, and the loss of allelic richness. In contrast, a bottleneck identified in *A*. *meeki* is attributed to the rapid loss of allelic diversity; however, the R_ST_ value and the BSP analysis (in the HPD range) indicates that the bottleneck detected by IAM were overestimated. The population dynamics of *A*. *meeki* can be explained by the variations in nucleotide diversity and proportion of segregating sites (π < Θs), which is interpreted as a recent population reduction followed by expansion, as demonstrated by the estimated value of D_T_ = -0.86 and non-significant positive values of Fu’s FS estimates [101–103].

Based in our results, we conclude that natural processes, such as geologic events and climate fluctuations, are the main factors responsible for the isolation of *A*. *diazi* from *A*. *meeki* during the Holocene, not enough time to diverge and showing an incomplete lineages sorting.

### Allotoca catarinae

Divergence times and isolation of *A*. *catarinae* from *A*. *diazi* and *A*. *meeki* was calculated to be within the past 8000–700 years and 8000–800 years, respectively. The peak of probability for isolation of *A*. *caratinae* was 1900 years ago. The low number of native species fish shared [12,88], and the geological and climatic records not support the connection and disconnection of the region in ancient periods: as the formation of Cupatitzio River ~30 mya [104,105], or in recent periods: as the activity and collapse of Tancítaro volcano 792 000±22 000 years ago [106,107], and the origin of Zirahuén Lake 17 000 years ago [92,93,108], contrasting the Álvarez hypothesis (1972) [21].

We propose that the existence of this taxon in the Cupatitzio River could be the result of a pre-Hispanic introduction, causing a founder effect, which resulted in rapid fixation of a single Cyt*b* haplotype in populations, along with a loss of genetic diversity. The lack of shared haplotypes between Cupatitzio populations and Pátzcuaro-Zirahuén populations cloud this interpretation, but the specimens sampled in the Pátzcuaro basin were from a small spring 10 km east of the lake and represent the only known local population of the species, which has been extirpated form the main basin [5]. We speculate that the ancestral haplotype introduced into Cupatitzio came from the main basin, and ancestral haplotype is lost. The haplotypes collected from the small spring (50 m^2^ approximately) were isolated from the main basin, and the genetic drift fixed rare haplotypes. In addition, null genetic diversity was found in *A*. *catarinae*, associated with recent bottlenecks. *Allotoca catarinae* is the only one in which bottlenecks were detected in several models of evolution, and also supported with the BSP analysis. These bottlenecks drive in all cases to low heterozygosity (F_IS_ = 0.151).

Relevant anthropogenic events in the region of Lakes Pátzcuaro and Zirahuén and the Cupatitzio River are congruent with our estimated divergence times. There is evidence that fishing was a major activity among the P'urhépecha. Fish, specifically goodeines and atherinopsids, had importance as food, even today goodeids are important part of the food supply for natives in the region, as well as culturally, in all socioeconomic strata, especially for Cazonci, the supreme ruler of the P’urhépecha state. Offerings of fish found in tombs at the archaeological site Uricho southwest of Lake Pátzcuaro, dating to the Late and Epi-Classic periods (~500–900 A.D.) [7,8].

The “islander”, “Chichimeca” and the “Coringuaro”, considered as first human groups to settle in the region of Uruapan and south of Michoacán, probably had influence on the management of aquatic resources and translocation of species may have occurred [109,110]. However, the information about their biological impact is less well documented. The rise of the P'urhépecha culture occurred around 1350, during the formation of the empire, with establishment of new villages, and species translocations possibly took place [109–112]. The expansion of the P'urhépecha Empire occurred during the second period with establishment around 1400 (second period) and 1450–1530 (third period) (S3 Fig). During these periods, settlements ranged from the region of the La Palma River, to the Marqués River region, in the Balsas Depression. Evidence from the P'urhépechas settlements in the Cupatitzio River subbasin and the Tepalcatepec-Balsas River suggests that, in this period, *A*.*diazi* translocation may have occurred. We conclude that the biogeographic history of *A*. *catarinae* was possibly determined by a founder effect mediated by pre-Hispanics in the past 3000 years, and taking into account this inference we cannot rule out species translocations in Pátzcuaro and Zirahuén lakes.

### Conservation implications

The factors involved in the genetic diversity and demographic history of *A*. *diazi* and *A*. *meeki*, were not only the founder effect (*A*. *meeki*) and climate fluctuations (*A*. *diazi*), but also anthropogenic activity [93–99].

The two OCU’s proposals (Pátzcuaro and Zirahuén lakes) are areas with a high degree of alteration, including eutrophication, introduction of exotic species, drought, and overfishing [11,12,22,24,113–115], which led to the extirpation of *A*. *diazi* from the main Pátzcuaro basin and *A*. *meeki* from Zirahuén Lake. Neither Pátzcuaro nor Zirahuén are designated protected areas. In addition, the results of the future ecological niche modeling for *A*. *diazi and A*. *meeki* show a reduction of potential habitats questioning the survival of the species, taking into account its genetic vulnerability to climate fluctuations and human activity, which indicates the necessity of the implementation of effective conservation and management strategies of this micro-endemic species with a long history of economic and cultural importance to the lacustrine settlements.

## Supporting Information

S1 Fig Number of groups (K) to explain the genetic structure by ΔK criteria. (EPS)

S2 Fig Marginal distribution with Isolation-with-Migration model of three scenarios of ancestry based on mitochondrial Cyt*b* gene and microsatellite loci (ESS>100 000). (EPS)

S3 Fig Extension of the P’urhépecha Empire during the period ca. 1450–1520 A.D. **Modified from [1]**.
(TIF)

S4 FigDemographic history of the members Allotoca diazi complex using Bayesian skyline plots from Cytb sequences.Dotted lines represent the location of the upper bound (Max), the mean ($\bar{X}$) and lower bound of the HPD≥95%.(EPS)

S1 Table Localities and sample size. (DOC)

S2 Table Selected polymorphic microsatellite loci. (DOC)

S3 Table Evolutionary substitution model from mitochondrial Cytb gene by Akaike Information Criterion (AIC). -lnL = log likelihood, Ti = Transitions, Tv = Transversions.
(DOC)

S4 Table Variables included in ecological niche models. RM = Regularization multiplier value selected according to the of AUC value.
(DOC)

S5 Table Estimated allelic diversity by locus and species. n = sample size, N_a_ = mean of alleles, N_ae_ = mean number of effective alleles, P_an_ = null allele proportion, LD = linkage disequilibrium test with significant value after Bonferroni correction (* = *P* > 0.05).
(DOC)

S6 Table Genetic structure based on R_ST_ values compared with F_ST_ of permutation analysis. H_o_ R_ST_ = *p*R_ST_, ns = non-significant, *significant (*P* < 0.05) after Bonferroni correction, R_ST_ = observed values, *p*R_ST_ = expected values, IC = 95% confidence interval.
(DOC)

S7 Table Genetic structure inferred via Analysis of Molecular Variance AMOVA based on microsatellite data. (DOC)
