# Supplementary figures and images for: Evolutionary History of the Live-Bearing Endemic Allotoca diazi Species Complex (Actinopterygii, Goodeinae): Evidence of Founder Effect Events in the Mexican Pre-Hispanic Period
Source: PLoS One. 2015 May 6;10(5):e0124138. doi: 10.1371/journal.pone.0124138 (PMC4422623; doi:10.1371/journal.pone.0124138)

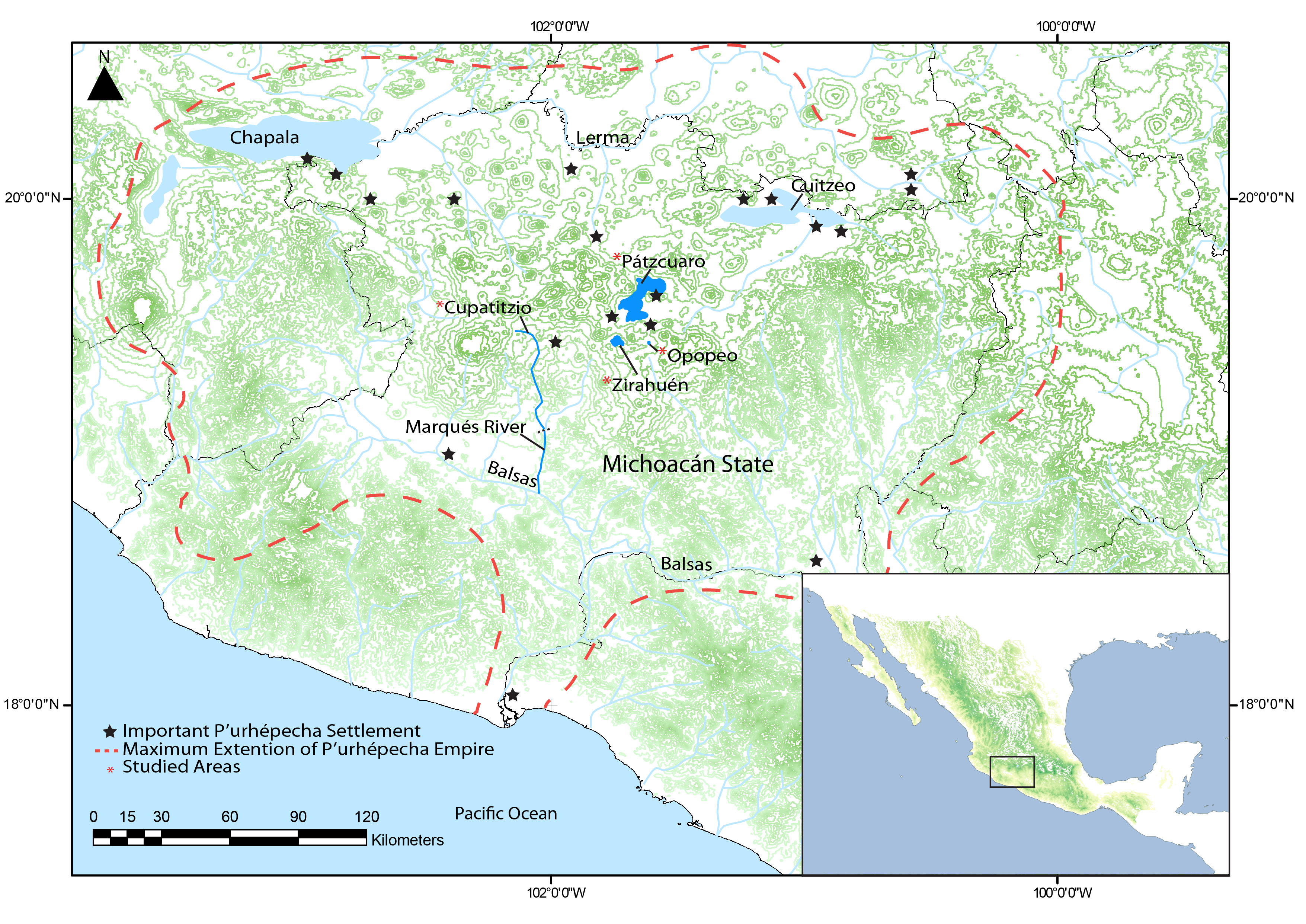

Supplement: S3 Fig — Modified from [1]. (TIF) [file pone.0124138.s003.tif]
