# Supplementary material for: Evolutionary History of the Live-Bearing Endemic Allotoca diazi Species Complex (Actinopterygii, Goodeinae): Evidence of Founder Effect Events in the Mexican Pre-Hispanic Period
Source: PLoS One. 2015 May 6;10(5):e0124138. doi: 10.1371/journal.pone.0124138 (PMC4422623; doi:10.1371/journal.pone.0124138)
Supplement: S1 Table — (DOC) [file pone.0124138.s005.doc]

**Table S1** Localities and sample size

| Species | n Cyt*b* | n Microsatellites | Localities | GenBank accession number | Coordinates |
| --- | --- | --- | --- | --- | --- |
| *A. catarinae* | 13 | 9 | Santa Catarina Dam | KJ776467-KJ776479 | N 19°25’18.39’’  W 102°00’59.9’’ |
|  | 15 | 15 | Urban Ecological Park | KJ776493-KJ776507 | N 19°23’23.7’’  W 102°00’51.8’’ |
|  | 11 | 9 | El Sauco Stream | KJ776508-KJ776518 | N 19º20’58.4’’  W 104º04’50.6’’ |
|  | 13 | 9 | Cupatitzio Dam | KJ776480-KJ776492 | N 19°20’33.4’’  W 102°08’00.8’’ |
| *A. diazi* |  |  | Pátzcuaro Lake (possibly extirpated) |  |  |
|  | 35 | 37 | Chapultepec Spring | KJ776519- KJ776553 | N 20º34’19.1’’  W 101º31’29.3’’ |
| *A. meeki* |  |  | Zirahuén Lake (extirpated) |  | N 19º27’00’’  W 101º45’00’’ |
|  | 22 | 22 | Opopeo | KJ776554- KJ776575 | N 19º24’22’’  W 101º36’13.5’’ |
