# Supplementary material for: Evolutionary History of the Live-Bearing Endemic Allotoca diazi Species Complex (Actinopterygii, Goodeinae): Evidence of Founder Effect Events in the Mexican Pre-Hispanic Period
Source: PLoS One. 2015 May 6;10(5):e0124138. doi: 10.1371/journal.pone.0124138 (PMC4422623; doi:10.1371/journal.pone.0124138)
Supplement: S2 Table — (DOC) [file pone.0124138.s006.doc]

**Table S2** Selected polymorphic microsatellite loci

| Locus | Repetition | Primer sequence | Length |
| --- | --- | --- | --- |
| ZT1.6 | (TG)_21_ | F GCACCTCCTAATACCTCACA  R AAGCAGCAGTACTGTGAGCT | 240-306 |
| ZT1.7 | (GGAT)_12_ | F TTGCTATATTGTCAGTCT  R ACTGAAATACTGTTTGTGT | 130-250 |
| ZT1.9 | (GGAT)_30_ | F CTGATGGTTTGAATCTATGC  R TGAAGAAGCTGTCTGAGAGG | 392-487 |
| XC18 | (CA)_14_ | F AGGTCATCCGGAGAAAGGT  R TTTCACAATCAAACCCCATGT | 519-625 |
| XC25 | (AC)_16_ | F CCTATTTGGGCTCCTGCTG  R CTTTCTTCCGTCCGGACTCT | 178-196 |
| AS2 | (AC)_10_G(CA)_22_ | F CGAAAGCTGTTGTCCTCCT  R TTGTGATGCAAAAGCAGTCGT | 220-275 |
| IW196 | (CA)_13_ | F GGGGAGACAGCAAATGAGAA  R TATAGCGGGGCTTTTCACAC | 197-251 |
