# Supplementary material for: Evolutionary History of the Live-Bearing Endemic Allotoca diazi Species Complex (Actinopterygii, Goodeinae): Evidence of Founder Effect Events in the Mexican Pre-Hispanic Period
Source: PLoS One. 2015 May 6;10(5):e0124138. doi: 10.1371/journal.pone.0124138 (PMC4422623; doi:10.1371/journal.pone.0124138)
Supplement: S3 Table — -lnL = log likelihood, Ti = Transitions, Tv = Transversions. (DOC) [file pone.0124138.s007.doc]

**Table S3** Evolutionary substitution model from mitochondrial Cytb gene by Akaike Information Criterion (AIC)

| Criterion | Position | Evolutionary Model | -lnL | Substitution rate model Ti/Tv | Nucleotide empiric base frequency | Gamma distribution | Invariable sites proportion |
| --- | --- | --- | --- | --- | --- | --- | --- |
| AIC | complete fragment | TIM+I+G | -1702.006 | -- | *A=*0.2476  *C=*0.2928  *G*=0.1349  *T*=0.3248 | 0.1490 | 0.9430 |

-lnL=log likelihood, Ti=Transitions, Tv=Transversions.
