# Supplementary material for: Evolutionary History of the Live-Bearing Endemic Allotoca diazi Species Complex (Actinopterygii, Goodeinae): Evidence of Founder Effect Events in the Mexican Pre-Hispanic Period
Source: PLoS One. 2015 May 6;10(5):e0124138. doi: 10.1371/journal.pone.0124138 (PMC4422623; doi:10.1371/journal.pone.0124138)
Supplement: S4 Table — RM = Regularization multiplier value selected according to the of AUC value. (DOC) [file pone.0124138.s008.doc]

**Table S4** Variables included in ecological niche models

| Species | Period in years ~ | RM | BIOvariables included | AUC |
| --- | --- | --- | --- | --- |
| *A. diazi* | Present | 0.01 | 1-15 and 17-19 | 0.876 |
|  | 2041-2060 | 0.01 | 7, 10, 14 and 19 | 0.940 |
|  | 2061-2080 | 0.01 | 5, 10, 11, 12, and 15 | 0.986 |
| *A. meeki* | Present | 1 | 1-15 and 17-19 | 0.932 |
|  | 2041-2060 | 0.01 | 5, 7, 9 and 19 | 0.965 |
|  | 2061-2080 | 0.01 | 5, 9, 10 and 19 | 0.995 |

RM= Regularization multiplier value selected according to the of AUC value
