# Supplementary material for: Evolutionary History of the Live-Bearing Endemic Allotoca diazi Species Complex (Actinopterygii, Goodeinae): Evidence of Founder Effect Events in the Mexican Pre-Hispanic Period
Source: PLoS One. 2015 May 6;10(5):e0124138. doi: 10.1371/journal.pone.0124138 (PMC4422623; doi:10.1371/journal.pone.0124138)
Supplement: S5 Table — n = sample size, Na = mean of alleles, Nae = mean number of effective alleles, Pan = null allele proportion, LD = linkage disequilibrium test with significant value after Bonferroni correction (* = P > 0.05). (DOC) [file pone.0124138.s009.doc]

**Table S5** Estimated allelic diversity by locus and species

| Locus |  | N_a_ | N_ae_ | P_an_ | LD |
| --- | --- | --- | --- | --- | --- |
| XC18 |  | 14.0±3.6 | 7,6±0.9 | 0.05 | -- |
| ZT1.6 |  | 10.0±3.6 | 5.9.0±3.3 | 0.04 | -- |
| ZT1.7 |  | 12.0±2.0 | 7.0±0.3 | 0.04 | -- |
| IW196 |  | 9.3±2.1 | 4.4±2.1 | 0.06 | -- |
| XC25 |  | 5.0±3.0 | 2.0±0.8 | 0.07 | -- |
| AS2 |  | 7.3±1.5 | 5.0±1.6 | 0.12 | -- |
| ZT1.9 |  | 15.0±1.7 | 8.6±3.3 | 0.07 | -- |
| Total |  | 10.4±3.5 | 5.8±2.2 | 0.06±0.03 |  |
| Species | n | N_a_ | N_ae_ | Pna | DL |
| *A. diazi* | 35 | 9.9±3.0 | 4.9±1.8 | 0.10 | AS2-ZT1.9* |
| *A. meeki* | 22 | 11.3±2.3 | 6.9±2.1 | 0.01 | -- |
| *A. catarinae* | 52 | 10.0±6.3 | 5.5±3.9 | 0.05 | -- |
| Total | 96 | 10.4±0.8 | 6.2±1.0 |  |  |

n=sample size, N_a_=mean of alleles, N_ae_=mean number of effective alleles, P_an_=null allele proportion, LD=linkage disequilibrium test with significant value after Bonferroni correction (*=*P* > 0.05).
