# Supplementary material for: Evolutionary History of the Live-Bearing Endemic Allotoca diazi Species Complex (Actinopterygii, Goodeinae): Evidence of Founder Effect Events in the Mexican Pre-Hispanic Period
Source: PLoS One. 2015 May 6;10(5):e0124138. doi: 10.1371/journal.pone.0124138 (PMC4422623; doi:10.1371/journal.pone.0124138)
Supplement: S6 Table — Ho RST = pRST, ns = non-significant, *significant (P < 0.05) after Bonferroni correction, RST = observed values, pRST = expected values, IC = 95% confidence interval. (DOC) [file pone.0124138.s010.doc]

**Table S6** Genetic structure based on R_ST_ values compared with F_ST_ of permutation analysis

| Locus | R_ST_ | *p*R_ST_ (95% I. C.) | F_ST_ |
| --- | --- | --- | --- |
| XC18 | 0.072 ns | 0.095 (-0.015-0.338) | 0.102* |
| ZT1.6 | 0.527 ns | 0.139 (-0.012-0.441) | 0.146* |
| ZT1.7 | 0.266 ns | 0.088 (-0.014-0.310) | 0.091* |
| IW196 | 0.228 ns | 0.162 (-0.015-0.513) | 0.189* |
| XC25 | 0.158 ns | 0.083 (-0.016-0.230) | 0.088* |
| AS2 | -0.001 ns | 0.113 (-0.015-0.356) | 0.113* |
| ZT1.9 | 0.141 ns | 0.048 (-0.018-0.183) | 0.051* |
| All loci | 0.178 ns | 0.099 (0.026-0.205) | 0.113* |

H_o_ R_ST_=*p*R_ST_, ns=non-significant, *significant (*P* < 0.05) after Bonferroni correction, R_ST_=observed values, *p*R_ST_=expected values, IC=95% confidence interval
