# Supplementary material for: Evolutionary History of the Live-Bearing Endemic Allotoca diazi Species Complex (Actinopterygii, Goodeinae): Evidence of Founder Effect Events in the Mexican Pre-Hispanic Period
Source: PLoS One. 2015 May 6;10(5):e0124138. doi: 10.1371/journal.pone.0124138 (PMC4422623; doi:10.1371/journal.pone.0124138)
Supplement: S7 Table — (DOC) [file pone.0124138.s011.doc]

**Table S7** Genetic structure inferred via Analysis of Molecular Variance AMOVA based on microsatellite data

| Groups | FST | FSC | FCT | % among groups | % within groups | *P* |
| --- | --- | --- | --- | --- | --- | --- |
| (*A. diazi*) (*A. meeki*) (*A. catarinae*) | 0.113 | - | - | - | - | <0.05 |
| (*A. diazi*, *A. meeki*) *A. catarinae* | 0.117 | 0.086 | 0.038 | 3.27 | 8.28 | 0.33 |
| *A. diazi* (*A. meeki*, *A. catarinae*) | 0.118 | 0.106 | 0.011 | 1.09 | 10.43 | 0.67 |
| (*A. diazi*, *A. catarinae*) *A. meeki* | 0.120 | 0.127 | -0.036 | 3.55 | 13.19 | 1 |
